# Association of Intravenous Potassium and Magnesium Administration With Spontaneous Conversion of Atrial Fibrillation and Atrial Flutter in the Emergency Department

**DOI:** 10.1001/jamanetworkopen.2022.37234

**Published:** 2022-10-19

**Authors:** Filippo Cacioppo, Denise Reisenbauer, Harald Herkner, Julia Oppenauer, Nikola Schuetz, Jan Niederdoeckl, Sebastian Schnaubelt, Sophie Gupta, Martin Lutnik, Alexander Simon, Alexander O. Spiel, Nina Buchtele, Hans Domanovits, Anton N. Laggner, Michael Schwameis

**Affiliations:** 1 Department of Emergency Medicine, Medical University of Vienna, Vienna, Austria; 2 Department of Clinical Pharmacology, Medical University of Vienna, Vienna, Austria; 3 Department of Emergency Medicine, Clinic Ottakring, Vienna, Austria; 4 Department of Medicine I, 13i2, Medical University of Vienna, Vienna, Austria

## Abstract

**Importance:**

Whether the simultaneous intravenous administration of potassium and magnesium is associated with the probability of spontaneous conversion to sinus rhythm (SCV) in the acute treatment of atrial fibrillation (AF) and atrial flutter (AFL) is unknown.

**Objective:**

To assess potassium and magnesium administration and SCV probability in AF and AFL in the emergency department.

**Design, Setting, and Participants:**

A registry-based cohort study was conducted in the Department of Emergency Medicine of the Medical University of Vienna, Austria. All consecutive patients with AF or AFL were screened between February 6, 2009, and February 16, 2020.

**Interventions:**

Intravenous administration of potassium, 24 mEq, and magnesium, 145.8 mg.

**Main Outcomes and Measures:**

The primary outcome was the probability of SCV during the patient’s stay in the emergency department. Multivariable cluster-adjusted logistic regression was used to estimate the association between potassium and magnesium administration and the probability of SCV.

**Results:**

A total of 2546 episodes of nonpermanent AF (median patient age, 68 [IQR, 58-75] years, 1411 [55.4%] men) and 573 episodes of nonpermanent AFL (median patient age, 68 [IQR, 58-75] years; 332 [57.9%] men) were observed. In AF episodes, intravenous potassium and magnesium administration vs no administration was associated with increased odds of SCV (19.2% vs 10.4%; odds ratio [OR], 1.98; 95% CI, 1.53-2.57). In AFL episodes, in contrast, no association was noted for the probability of SCV with potassium and magnesium vs no administration (13.0% vs 12.5%; OR, 1.05; 95% CI, 0.65-1.69).

**Conclusions and Relevance:**

The findings of this registry-based cohort study on intravenous administration of potassium and magnesium suggest an increased probability of SCV in nonpermanent AF, but not AFL, during a patients’ stay in the emergency department.

## Key Points

**Question:**

Is the administration of potassium and magnesium associated with the probability of spontaneous conversion of nonpermanent atrial fibrillation and atrial flutter in the emergency department?

**Findings:**

In this cohort study that included 2546 episodes of atrial fibrillation and 573 episodes of atrial flutter, administration of potassium and magnesium was associated with higher rates of spontaneous conversion in atrial fibrillation vs no administration (19.2% vs 10.4%) during patients’ stay in the emergency department. However, there was no association with atrial flutter (13.0% vs 12.5%).

**Meaning:**

The findings of this study suggest that administration of potassium and magnesium might lessen the need for antiarrhythmic therapy and the potential adverse effects in patients with nonpermanent atrial fibrillation.

## Teaser

This cohort study examines the association between administration of combined intravenous potassium and magnesium and spontaneous conversion to sinus rhythm among patients with atrial fibrillation or atrial flutter in the emergency department.

## Introduction

Atrial fibrillation (AF) is becoming an increasing burden for health care systems worldwide owing to population aging.^1,2^ Pharmacologic and electrical cardioversion are common therapies in emergency departments (EDs), especially for highly symptomatic patients. Each intervention has specific risks, and neither is considered cost-effective owing to frequent recurrence of AF.^3^ In addition, AF often terminates spontaneously.^4^ Thus, implementation of wait-and-watch strategies combined with scoring systems to estimate the probability of spontaneous conversion to sinus rhythm (SCV) might be an innovative approach to avoid unnecessary antiarrhythmic therapy and its potential adverse effects.^5^ Moreover, evidence suggests that hypokalemia and hypomagnesemia contribute to AF development.^6,7^ Thus, the administration of potassium and magnesium could be a reasonable strategy to improve SCV rates.

Hypokalemia results in intracellular calcium overload and prolonged action potential duration, prompting early afterdepolarizations.^8^ Findings from an animal study suggests that low potassium levels also may trigger delayed afterdepolarizations and that both early and delayed afterdepolarizations are critical factors in AF development.^9^ Magnesium regulates cardiac cation channels. Changes in magnesium concentration thus may alter cellular excitability and increase the risk of cardiac arrhythmias.^10^ Furthermore, administration of magnesium has been suggested to provoke antiarrhythmic effects by prolonging atrial refractory periods.^11,12^

To date, data on the administration of potassium and magnesium in patients with AF and atrial flutter (AFL) are limited. In this study, we analyzed the use of combined intravenous administration of potassium and magnesium for the probability of SCV in patients with nonpermanent AF and AFL who were treated at an academic ED.

## Methods

This registry-based cohort study is based on an observational arrhythmia registry operated by the Department of Emergency Medicine at Vienna General Hospital, Austria, a 1700-bed tertiary care facility and university hospital of the Medical University of Vienna. The ED comprises outpatient, intermediate, and critical care units, covering up to 85 000 patients per year. Both AF and AFL are treated in accordance with current European Society of Cardiology guidelines.^13,14^

The registry includes all consecutive adult patients (age ≥18 years) with supraventricular tachycardia confirmed by 12-lead electrocardiography who are treated at the ED. Study fellows document patient data, including age, sex, vital signs, electrocardiographic rhythm, blood gas analyses, laboratory test values, daily medication, drugs administered during the ED visit, and course of treatment. Laboratory values are imported automatically from the hospital's laboratory system. For this study, all patients aged 18 years or older presenting to the ED with AF or AFL between February 6, 2009, and February 16, 2020, were eligible for inclusion, regardless of electrolyte levels, organ function, pregnancy, or long-term oral antiarrhythmic therapy. Only patients with permanent AF or AFL were excluded. The ethics committee of the Medical University of Vienna approved use of the registry. Data reporting follows the Strengthening the Reporting of Observational Studies in Epidemiology (STROBE) reporting guideline.^15^

### Standard Treatment Regimen

Current guidelines do not provide treatment recommendations for or against electrolyte administration in AF or AFL.^13,14^ Therefore, the decision to administer potassium or magnesium is at the discretion of the treating physician and usually based on the potassium level measured in the venous blood gas analysis on admission to the ED. For electrolyte administration, a prefabricated electrolyte infusion (Elozell “spezial”) containing potassium, 24 mEq (to convert to millimoles, multiply by 1), and magnesium, 145.8 mg (to convert to millimoles, multiply by 0.04115), per 250-mL infusion bag, usually combined with 500 mL of balanced crystalloid fluid (containing potassium, 2.5 mEq, and magnesium, 18.2 mg), is administered for 90 minutes. In patients experiencing pain at the injection site, the infusion rate is reduced until freedom from pain is achieved. There is no standardized observation period after the start of the infusion, and patients may proceed immediately to rhythm or rate control therapy.

### Potassium and Magnesium Values

For statistical analysis, we used plasma potassium and plasma magnesium levels obtained on admission and measured by the ISO 15189:2012-certified Department of Laboratory Medicine at Vienna General Hospital (potassium reference range: 3.50-5.10 mEq/L [to convert to millimoles per liter, multiply by 1], magnesium reference range: 1.60-2.60 mg/dL [to convert to millimoles per liter, multiply by 0.4114]). Of 145 episodes for which plasma potassium levels were not available, 129 had potassium level measurements from venous blood gas analysis on admission, which were used instead.

### Spontaneous Conversion to Sinus Rhythm

Conversion to sinus rhythm was considered spontaneous if no attempt at pharmacologic rhythm control was made until conversion occurred. Pharmacologic rhythm control included treatment with ibutilide, vernakalant, sotalol, flecainide, propafenone, or amiodarone, irrespective of the dosage used. Conversion to sinus rhythm after an unsuccessful attempt at electrical cardioversion or following rate control with β-blockers, nondihydropyridine calcium channel blockers, or digitalis glycosides was considered spontaneous. Patients receiving long-term oral antiarrhythmic therapy were not excluded from the analysis because a relapse of AF or AFL was considered a treatment failure, and therefore no substantial effect on SCV in the ED was expected.

### Statistical Analysis

We present continuous data as median (IQR) and categorical data as absolute counts and relative frequencies. Multivariable cluster-adjusted logistic regression was used to estimate the probability of SCV associated with potassium and magnesium administration.

We present the outcomes as odds ratios (ORs) with 95% CIs. We used the Wald test to analyze the H_0_ (OR, 1) and the likelihood ratio test for assessing deviation from linearity or interactions (*P* value for interaction).

We developed directed acyclic graphs (DAGs) with multivariable regression analysis and subsequent change-in-estimate procedures (eMethods, eFigures 1-3, and eTable in the Supplement). We constructed a framework to identify potential confounders of the outcomes using DAGitty software, version 3.0.^16^ Based on the associations assumptions and role allocations laid out in the DAG, we subsequently performed a change-in-estimate procedure using a collapsible estimator (risk ratio with 95% CI) to make further variable adjustments. We estimated the change in the baseline estimate for outcome associated with potassium and magnesium administration when adjusted for other possibly related variables including C-reactive protein, heart rate, rate control medication, administration of diazepam in the ED, glomerular filtration rate (representing kidney function and calculated using the Chronic Kidney Disease Epidemiology Collaboration formula), arterial hypertension, pH, diabetes, coronary artery disease, intake of angiotensin II receptor blockers or angiotensin enzyme-converting inhibitors, sex, age, de novo AF, baseline calcium level, baseline potassium level, baseline magnesium level, heart failure, or onset of AF. According to the DAG change-in-estimate procedure, the most parsimonious regression model had to adjust only for baseline potassium levels. However, because residual confounding can never be completely ruled out (as with all statistical methods that use observational data), apart from baseline potassium levels, the final analysis included further variables considered relevant from a clinical perspective. These variables were baseline magnesium levels, onset of AF or AFL, heart failure, diabetes, arterial hypertension, intake of either angiotensin II receptor blockers or angiotensin enzyme-converting inhibitors, and kidney function. Because the outcome of the exposure (potassium and magnesium administration) was not constant across baseline levels of potassium and magnesium levels and the onset of AF and AFL, we handled these variables by stratification (potassium level strata: <3.5, 3.5-3.99, 4.0-4.49, ≥4.5 mEq/L; magnesium level strata: <1.87, 1.87-2.00, 2.01-2.12, ≥2.13 mg/dL; onset of AF or AFL strata: <48 hours, ≥48 hours, unknown). Using the remaining variables, multivariable adjustment was performed.

Some patients in our sample had repeated visits to the ED. Thus, to allow for the resulting panel structure of our data, we used cluster-robust methods with patient identification number from the hospital documentation system. Every episode documented in the registry has a unique number. In case the same patient presents twice to the ED, both episodes have different episode numbers but the same patient identification number as the cluster identifier. We used complete case sets and did not use missing data imputation methods. The level of the association between baseline potassium and baseline magnesium levels was estimated using the Pearson correlation coefficient.

We used Microsoft Excel 16, Stata 16 (StataCorp LLC), DAGitty software, version 3.0, and Prism 9 for data management, analysis, and graphic design. A 2-sided *P* value <.05 was considered statistically significant.

## Results

**Figure 1.**
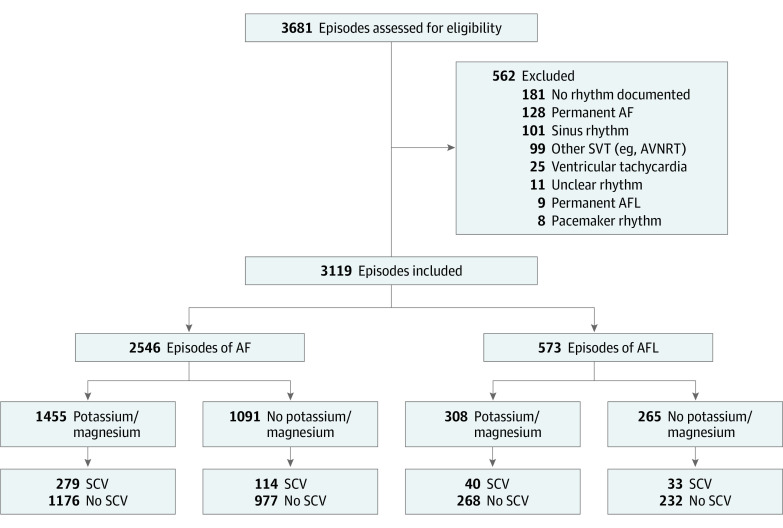
Study Flowchart Of 3681 episodes screened, 3119 episodes of atrial fibrillation (AF) (2546 [81.6%]) and atrial flutter (AFL) (573 [18.4%]) were included in the final analysis. Combined intravenous potassium and magnesium was administered in 1763 episodes. AVNRT, atrioventricular nodal reentry tachycardia; SCV, spontaneous conversion to sinus rhythm; SVT, supraventricular tachycardia.

Of 3681 screened AF and AFL episodes, 3119 episodes were analyzed (AF: 2546 [81.6%] episodes, median [IQR] age, 68 [58-75] years, 1411 [55.4%] men, 1135 [44.6%] women; AFL: 573 [18.4%] episodes, median age, 68 [IQR, 58-75] years, 332 [57.9%] men, 241 [42.1%] women) (Figure 1). Intravenous potassium and magnesium were administered in 1763 (56.5%) episodes. The median amount of administered potassium and magnesium (Elozell “spezial”) was 1 infusion bag in patients with both AF and AFL. The median duration of stay in the ED was 6.4 (IQR, 3.9-11.6) hours for patients with AF and 6.1 (IQR, 3.9-11.8) hours for patients with AFL. During the stay in the ED, SCV occurred in 15.4% (n = 393) of AF episodes and 12.7% (n = 73) of AFL episodes. Table 1 and Table 2 present baseline characteristics of patients regarding potassium and magnesium administration. No relevant correlation between baseline potassium and magnesium levels was found (*r* = 0.10; 95% CI, 0.07-0.14).

**Table 1.** Demographic Characteristics and Laboratory Findings of Patients With Atrial Fibrillation and Atrial Flutter Episodes

Characteristic	Atrial fibrillation				Atrial flutter			
	K‎/Mg administration, median (IQR) [n = 1455]	No. of episodes	No K‎/Mg administration, median (IQR) [n = 1091]	No. of episodes	K‎/Mg administration, median (IQR) [n = 308]	No. of episodes	No K‎/Mg administration, median (IQR) [n = 265]	No. of episodes
SCV, No. (%)	279 (19.2)	1455	114 (10.4)	1091	40 (13.0)	308	33 (12.5)	265
Duration until SCV, h	2.5 (1.38-4.26)	244	2.67 (1.11-4.67)	92	1.73 (0.8-2.38)	35	1.58 (0.83-2.74)	24
Age, y	66 (55-73)	1455	71 (62-78)	1091	66 (54-74)	308	70 (62-76)	265
Sex, No. (%)		1455		1091		308		265
Male	798 (54.8)		613 (56.2)		184 (59.7)		148 (55.8)	
Female	657 (45.2)		478 (43.8)		124 (40.3)		117 (44.2)	
Potassium, mEq/L	3.87 (3.63-4.11)	1454	4.22 (3.96-4.49)	1076	3.98 (3.75-4.2)	308	4.33 (4.06-4.6)	265
Magnesium, mg/dL	2.02 (1.87-2.14)	1387	2.02 (1.87-2.14)	1020	2.04 (1.90-2.14)	291	2.02 (1.87-2.14)	255
Onset, h	5 (2-12)	919	8 (3-24)	480	10 (3-29)	210	14 (5.5-48)	133
Onset unknown, No. (%)	536 (36.8)	1455	611 (56.0)	1091	98 (31.8)	308	132 (49.8)	265
Stay in ED, h	6.87 (4.33-12.03)	1400	5.93 (3.31-10.66)	1022	5.99 (3.91-9.94)	301	6.33 (3.96-13.89)	251
Highest heart rate, bpm	131 (113-147)	953	124 (101-142)	663	134 (121-146)	233	133 (122-150)	185
CHA2DS2-VASc score^a^	2 (1-4)	1403	3 (2-4)	1056	2 (1-4)	298	3 (2-4)	259
Laboratory findings								
pH	7.41 (7.38-7.44)	1373	7.39 (7.36-7.42)	992	7.41 (7.38-7.43)	293	7.39 (7.36-7.42)	244
Creatinine, mg/dL	0.95 (0.82-1.12)	1443	1.06 (0.88-1.29)	1057	0.98 (0.84-1.15)	305	1.04 (0.89-1.28)	263
NT-proBNP, pg/mL	651.6 (236.7-1880)	1284	1548 (631.7-3502)	876	1014 (415-2245.5)	280	1746 (668.3-3561)	229
Troponin T, ng/mL^b^	0.012 (0.007-0.022)	1149	0.017 (0.01-0.032)	769	0.013 (0.008-0.022)	249	0.018 (0.01-0.032)	212
CRP, mg/dL	0.31 (0.13-0.86)	1308	0.44 (0.17-1.31)	958	0.25 (0.13-0.78)	281	0.34 (0.15-1.38)	240
Lipase, U/L	32 (24-43)	1328	30 (22-44)	952	30 (24-44)	288	32 (22-47)	236
TSH, μIU/mL	1.85 (1.13-2.91)	1035	1.75 (1.03-2.76)	654	1.86 (1.2-2.82)	223	1.79 (1.13-2.9)	173
Hypothyroidism, No. (%)	2 (0.1)	1455	3 (0.3)	1091	0 (0.0)	308	0 (0.0)	265
Hyperthyroidism, No. (%)	27 (1.9)	1455	19 (1.7)	1091	2 (0.6)	308	3 (1.1)	265

Abbreviations: CHA2DS2-VASc, congestive heart failure, hypertension, age greater than or equal to 75 years, diabetes, prior stroke or transient ischemic attack, vascular disease, age 65 to 74 years, female; CRP, C-reactive protein; ED, emergency department; K/Mg, potassium and magnesium; NT-proBNP, N-terminal pro-b-type natriuretic peptide; SCV, spontaneous conversion to sinus rhythm; TSH, thyroid-stimulating hormone.

SI conversion factors: To convert creatinine to micromoles per liter, multiply by 88.4; CRP to milligrams per liter, multiply by 10; lipase to microkatals per liter, multiply by 0.0167; magnesium to millimoles per liter, multiply by 0.4114; NT-proBNP to nanograms per liter, multiply by 1; potassium to millimoles per liter, multiply by 1; troponin T to micrograms per liter, multiply by 1.

^a^ CHA2DS2-VASc score as indicator of the risk of stroke, with scores from 0 to 9.

^b^ During the study period, cardiac troponin T (nanograms per milliliter) was replaced by high-sensitive cardiac troponin T (nanograms per liter), and both are summarized as troponin T and presented as nanograms per milliliter.

**Table 2.** Classification of Atrial Fibrillation and Atrial Flutter, Treatment at the Emergency Department and Baseline Characteristics of Patients

Classification of AF/AFL episode	Episodes, No. (%)			
	Atrial fibrillation		Atrial flutter	
	K‎/Mg administration (n = 1455)	No K‎/Mg administration (n = 1091)	K‎/Mg administration (n = 308)	No K‎/Mg administration (n = 265)
Paroxysmal‎/persistent	1010 (69.4)	692 (63.4)	226 (73.4)	191 (72.1)
Long-standing	0	4 (0.4)	0	0
First episode	17 (1.2)	9 (0.8)	2 (0.6)	3 (1.1)
Second episode	6 (0.4)	15 (1.4)	1 (0.3)	1 (0.4)
Unknown	422 (29.0)	371 (34.0)	79 (25.6)	70 (26.4)
Treatment in the ED				
Rate control in ED	344 (23.6)	228 (20.9)	69 (22.4)	74 (27.9)
Rhythm control in ED^a^	1013 (69.6)	673 (61.7)	245 (79.5)	204 (77.0)
Crystalloid fluid, median (IQR), mL^b^	1000 (500-1000)	500 (500-1000)	1000 (500-1000)	500 (500-1000)
Oral diazepam	417 (28.7)	124 (11.4)	90 (29.2)	43 (16.2)
Cardiovascular conditions				
Arterial hypertension	931 (64.0)	720 (66.0)	174 (56.5)	172 (64.9)
Heart failure	262 (18.0)	256 (23.5)	42 (13.6)	57 (21.5)
Valvular heart disease	331 (22.7)	274 (25.1)	96 (31.2)	93 (35.1)
Coronary artery disease	199 (13.7)	238 (21.8)	41 (13.3)	60 (22.6)
Diabetes	189 (13.0)	177 (16.2)	40 (13.0)	45 (17.0)
Hyperlipidemia	471 (32.4)	337 (30.9)	113 (36.7)	111 (41.9)
Home medication				
Rate control agents	697 (47.9)	587 (53.8)	156 (50.6)	139 (52.5)
Antiarrhythmic agents	342 (23.5)	242 (22.2)	91 (29.5)	69 (26.0)
ACE inhibitors or ARBs	649 (44.6)	465 (42.6)	137 (44.5)	114 (43.0)
Diuretics	305 (21.0)	271 (24.8)	78 (25.3)	69 (26.0)
Calcium channel blockers	160 (11.0)	106 (9.7)	41 (13.3)	30 (11.3)
Thyroid hormone	171 (11.8)	117 (10.7)	32 (10.4)	36 (13.6)
Potassium	45 (3.1)	22 (2.0)	8 (2.6)	8 (3.0)
Magnesium	55 (3.8)	35 (3.2)	22 (7.1)	15 (5.7)

Abbreviations: ACE, angiotensin-converting enzyme; AF, atrial fibrillation; AFL, atrial flutter; ARBs, angiotensin II receptor blockers; ED, emergency department; K/Mg, potassium and magnesium.

^a^ Rhythm control refers to attempted pharmacologic or electrical cardioversion. Conversion to sinus rhythm after unsuccessful electrical cardioversion was considered a spontaneous conversion to sinus rhythm.

^b^ Data on crystalloid fluid administered in the ED were available in 1358 AF episodes with and 747 episodes without potassium and magnesium administration, and in 295 AFL episodes with and 213 episodes without potassium and magnesium administration.

**Figure 2.**
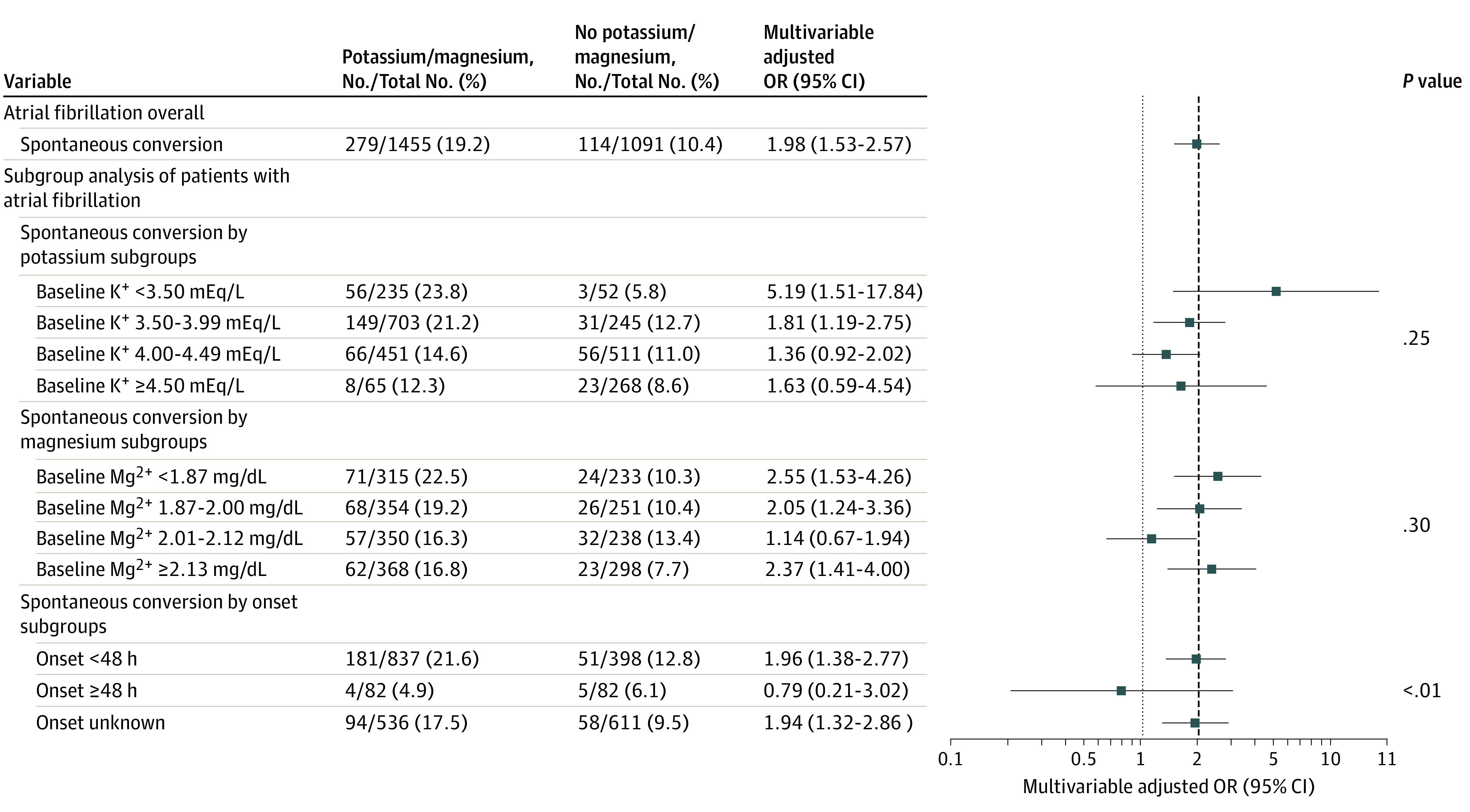
Effect Size Estimates for Spontaneous Conversion to Sinus Rhythm in Atrial Fibrillation Episodes and Atrial Fibrillation Subgroups In patients with atrial fibrillation, intravenous administration of potassium and magnesium were associated with a 419% increase in the odds of spontaneous conversion in those with a baseline potassium level less than 3.50 mEq/L and 81% in those with baseline potassium levels between 3.50 and 3.99 mEq/L. No association was seen in atrial fibrillation episodes for potassium levels between 4.00 and 4.49 mEq/L or greater than or equal to 4.50 mEq/L. Stratification by magnesium levels revealed consistency independent of baseline magnesium levels, except for the 2.01 to 2.12-mg/dL group. The models were adjusted for diabetes, heart failure, arterial hypertension, intake of either angiotensin II receptor blockers or angiotensin-converting enzyme inhibitors and glomerular filtration rate. *P* values refer to interactions between subgroups.

In AF episodes, intravenous administration of potassium and magnesium vs no administration was associated with increased odds of SCV (19.2% vs 10.4%; OR, 1.98; 95% CI, 1.53-2.57). Stratification for baseline potassium levels revealed that potassium and magnesium administration was associated with significantly higher odds of SCV in AF episodes with baseline potassium levels less than 3.50 mEq/L and 3.50 to 3.99 mEq/L. In contrast, no association was found with potassium levels greater than 3.99 mEq/L. Effect size estimates were largely constant across magnesium levels. In episodes with symptom onset greater than or equal to 48 hours, potassium and magnesium administration had no association with SCV probability (Figure 2).

**Figure 3.**
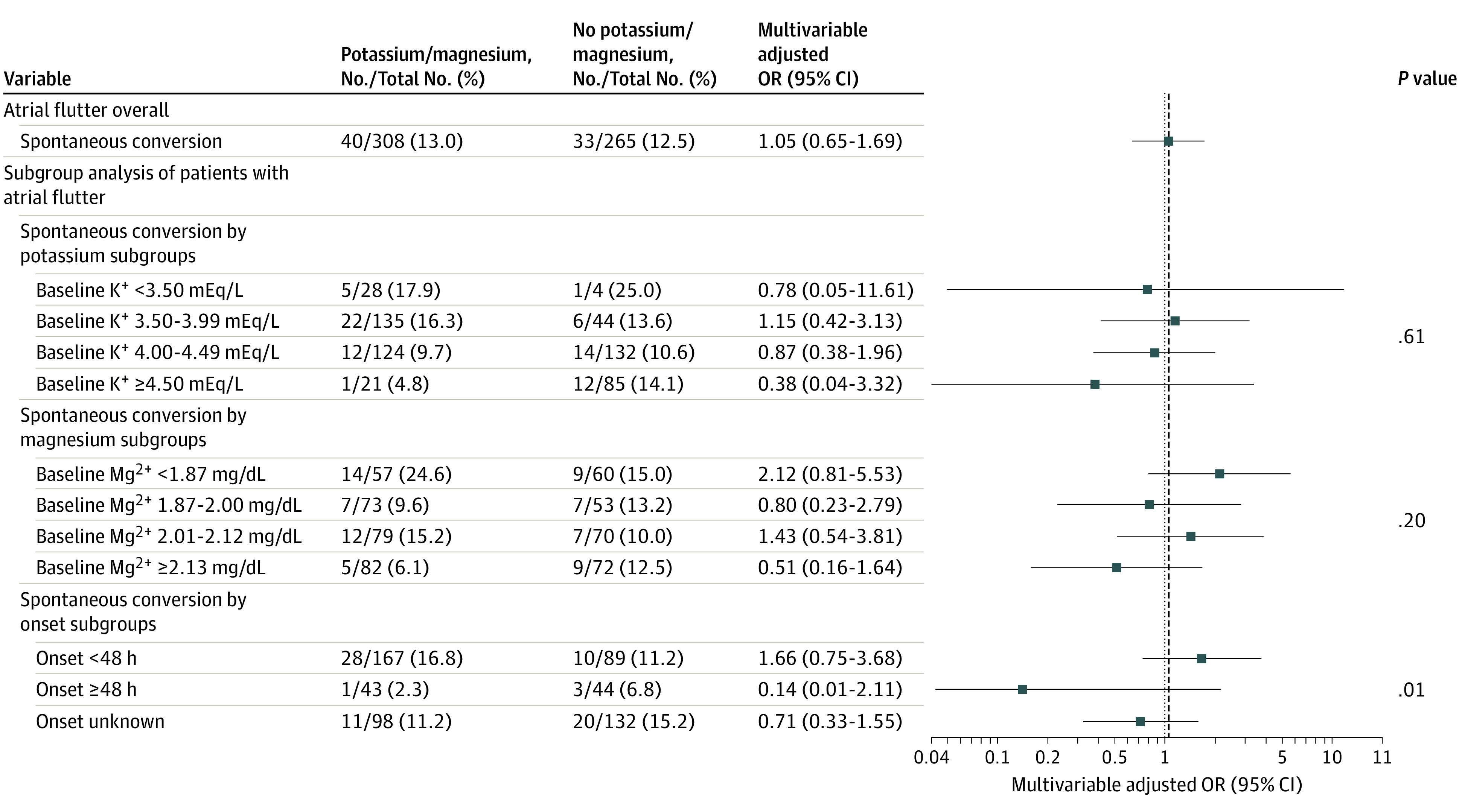
Effect Size Estimates for Spontaneous Conversion to Sinus Rhythm in Atrial Flutter Episodes and Atrial Flutter Subgroups The models were adjusted for diabetes, heart failure, arterial hypertension, intake of either angiotensin II receptor blockers or angiotensin-converting enzyme inhibitors, and glomerular filtration rate. *P* values refer to interactions between subgroups.

In episodes with AFL, intravenous potassium and magnesium administration vs no administration was not associated with the chances of SCV (13.0% vs 12.5%; OR, 1.05; 95% CI, 0.65-1.69), irrespective of baseline potassium or magnesium levels and symptom onset (Figure 3). A significant between-group difference was noted when comparing the association of intravenous potassium and magnesium administration with the overall SCV rates for AF and AFL (*P* = .02 for interaction).

## Discussion

In this registry-based study, intravenous administration of potassium and magnesium was associated with a higher probability of SCV in patients with AF, but it had no association with SCV probability in patients with AFL. To date, it is unclear whether potassium and magnesium administration might be reasonable in the acute treatment of AF and AFL, and although this intervention may be common practice in some EDs, it is not part of the treatment recommendations in current guidelines.^13,14,17,18^ Our findings suggest that intravenous potassium and magnesium administration may increase the chance of SCV in patients with AF with either hypokalemia or with plasma potassium levels in the range of 3.50 to 3.99 mEq/L. In patients with AFL, however, potassium and magnesium administration may not be associated with SCV probability.

In a placebo-controlled single-blind trial, Tazmini et al^19^ randomized 113 patients with a potassium level of less than or equal to 4 mEq/L and a symptom onset of less than or equal to 48 hours to receive either potassium chloride (added to 5% dextrose solution) or dextrose, 5%, only (placebo) as a continuous infusion. Conversion rates did not differ significantly between the 2 groups, which contradicts our results. However, Tazmini et al did not analyze AF and AFL separately, which might have diluted the effect of potassium administration on conversion probability. Furthermore, their sample size may not have been large enough to yield statistically significant results. A significant increase in conversion rates was observed in the subgroup of patients who received potassium at the highest infusion rate of 15 mEq/h, compared with those who received the placebo. The subgroup with the highest infusion rate may be best compared with our study patients, who typically received potassium at rates of 15 to 20 mEq/h.

The value of magnesium substitution for rhythm control in AF and AFL is likewise unclear. High-quality evidence from randomized clinical trials is lacking to support or oppose the administration of magnesium in nonpostoperative AF and AFL. Two previous meta-analyses yielded conflicting results.^20,21^ Whereas Onalan et al^20^ found magnesium to be more effective than calcium channel blockers, Ho et al^21^ found similar conversion rates between magnesium and placebo or antiarrhythmic drugs, including amiodarone or calcium channel blockers. The sample sizes and quality of the studies included in the 2 meta-analyses were limited.

In our study, we found a relatively constant association of potassium and magnesium administration with SCV across magnesium baseline levels. However, because hypomagnesemia may cause hypokalemia refractory to potassium substitution, coadministration of both electrolytes seems reasonable.^22,23^

In patients with a known onset of AF, we observed that intravenous administration of potassium and magnesium was associated with SCV only in patients with symptom onset of less than 48 hours, suggesting a time-dependent outcome. However, because only a limited number of patients with SCV had symptom onset greater than or equal to 48 hours, this finding warrants further investigation. The odds for SCV after potassium and magnesium administration were similar for AF onset of less than 48 hours before the intervention and unknown onsets, suggesting that numerous patients with unknown symptom onset likely had AF onset of less than 48 hours.

In patients with AFL, potassium and magnesium were not associated with SCV probability, perhaps because AFL differs from AF in its pathomechanism. Whereas AF is sustained by a complex composite of ectopic activity and multiple small electrical reentry circuits,^24^ the pathophysiologic characteristics of AFL are typically based on a fixed macro reentry.^25^ Thus, maintenance of AFL is probably less dependent on arrhythmogenic triggers, such as early afterdepolarizations, and hence might be less vulnerable to changes in electrolyte homeostasis and correction of electrolyte imbalances. Our data suggest that AF and AFL should be handled as 2 distinct disorders from a mechanistic and a therapeutic perspective.

The results of our study have no direct implications for clinical practice in the management of care for patients with AF or AFL in the ED. The findings are purely exploratory and hypothesis-generating but could potentially provide a rationale for an appropriate prospective trial.

### Strengths and Limitations

The strengths of the study are the clinical practice setting and the large number of episodes analyzed. We included all patients with AF or AFL who presented to the ED and were enrolled in the registry between February 2009 and February 2020 and excluded only patients with permanent AF or AFL to obtain a representative sample. We attempted to control for confounding by developing directed acyclic graphs with proper and transparent regression analysis and subsequent change-in-estimate procedures. We also used cluster-robust methods to allow for accurate interpretation at the patient level and to account for correlated response probabilities among patients who presented repeatedly to the ED.

This study has limitations. Bias owing to the cohort study design cannot be excluded. Because the time at risk was not standardized, bias due to competing risks from pharmacologic and electrical cardioversion must be considered. Selection bias may also affect the results. A systematic tendency to misclassify the risk factor or outcome seems unlikely but cannot be excluded. In addition, we cannot rule out the possibility of residual confounding, particularly confounding by indication, because unmeasured variables may have influenced the decision to administer potassium and magnesium.

Moreover, the registry documents the course of treatment at the ED but does not include data on rhythm control before ED admission. However, pill in-the-pocket approaches and pharmacologic rate control attempts by out-of-hospital emergency medical services are rare. In addition, the dosing regimen of intravenous potassium and magnesium is not standardized, and because we investigated the association of a combined potassium and magnesium solution with the probability of SCV, it is not possible to dissect the extent to which potassium and magnesium each contributed to the observed outcomes.

As with all statistical methods that use observational data, only measured variables can be considered, and residual confounding and bias cannot be eliminated. Therefore, appropriate caution is needed when interpreting our results. In addition, because of the smaller sample size of patients with AFL, our estimates are less reliable than those of the AF group. In this regard, all treatment decisions must be based on high-quality evidence from randomized clinical trials in which bias can be adequately accounted for. Nevertheless, considering the limitations inherent in an observational study, the present study may provide valuable data and rationale for the design and conduct of a randomized clinical trial to prospectively evaluate the effect of potassium and magnesium administration on the likelihood of SCV in nonpermanent AF.

## Conclusions

In this registry-based study, the intravenous administration of potassium and magnesium was associated with a higher probability of SCV in patients with nonpermanent AF and plasma potassium levels of less than 3.50 mEq/L and 3.50 to 3.99 mEq/L but not in patients with AFL. This approach might lessen the need for pharmacologic intervention and potential adverse effects caused by antiarrhythmic therapy. A randomized clinical trial is warranted to determine the efficacy and safety of such an approach.

## Notes

Supplement. **eMethods.** Statistical Analysis **eFigure 1.** Directed Acyclic Graph **eFigure 2.** Minimal Sufficient Variable Adjustment Set After Controlling for Biasing Paths **eTable.** Variables Included in the Change-in-Estimate Procedure **eFigure 3.** Changes in Baseline Estimate by Subtracting or Adding Possibly Confounding Variables
eReferences
