# Error in Figure 2

**DOI:** 10.1001/jamanetworkopen.2022.45392

**Published:** 2022-11-16

In the Original Investigation titled “Association of Intravenous Potassium and Magnesium Administration With Spontaneous Conversion of Atrial Fibrillation and Atrial Flutter in the Emergency Department,”^1^ published October 19, 2022, there was an error in Figure 2. The bold dashed line that appeared slightly to the right of the 2 value on the x-axis should have appeared in the position indicating “1.98.” This article has been corrected.^1^
