# Supplementary material for: Association of Intravenous Potassium and Magnesium Administration With Spontaneous Conversion of Atrial Fibrillation and Atrial Flutter in the Emergency Department
Source: JAMA Netw Open. 2022 Oct 19;5(10):e2237234. doi: 10.1001/jamanetworkopen.2022.37234 (PMC9582905; doi:10.1001/jamanetworkopen.2022.37234)
Supplement: Supplement. — eMethods. Statistical Analysis eFigure 1. Directed Acyclic Graph eFigure 2. Minimal Sufficient Variable Adjustment Set After Controlling for Biasing Paths eTable. Variables Included in the Change-in-Estimate Procedure eFigure 3. Changes in Baseline Estimate by Subtracting or Adding Possibly Confounding Variables eReferences [file jamanetwopen-e2237234-s001.pdf]

Supplementary Online Content

Cacioppo F, Reisenbauer D, Herkner H, et al. Association of intravenous potassium and magnesium administration with spontaneous conversion of atrial fibrillation and atrial flutter in the emergency department. *JAMA Netw Open*. 2022;5(10):e2237234. doi:10.1001/jamanetworkopen.2022.37234

eMethods. Statistical Analysis

eFigure 1. Directed Acyclic Graph

eFigure 2. Minimal Sufficient Variable Adjustment Set After Controlling for Biasing Paths

eTable. Variables Included in the Change-in-Estimate Procedure

eFigure 3. Changes in Baseline Estimate by Subtracting or Adding Possibly Confounding Variables

eReferences

This supplementary material has been provided by the authors to give readers additional information about their work.

© 2022 Cacioppo C et al. *JAMA Network Open.*

eMethods. Statistical Analysis

We attempted to control for confounding by employing the concept of causal inference from the counterfactual theory using directed acyclic graphs (DAGs) with proper and transparent regression analysis and subsequent change-in-estimate procedures.

We created a directed acyclic graph using DAGitty version 3.0 software [1] to visualize assumptions about the relationships between variables and to identify confounding.

We defined "potassium/magnesium administration" as the exposure variable (effect) and "spontaneous conversion to sinus rhythm" as the outcome variable (cause).

The remaining variables were handled as covariables and considered as a **collider** (a variable that is causally influenced by ≥ 2 variables, which must not be controlled for, as conditioning on colliders may introduce bias), a **mediator** (a descendant of the exposure and an ancestor of the outcome), which can be adjusted for, or a **confounder** (an ancestor of both the exposure and the outcome), which must be controlled for.

These variables were considered relevant, and the following variable assumptions were made:

1) Ancestors of "baseline potassium level" (which was considered the main driver of the exposure): "Oral potassium supplementation", "hemolysis", "endogenous catecholamine level", "insulin therapy", "vomiting/diarrhea", "pH", "renal tubular acidosis", "aldosterone", "diuretics" and "kidney disease (glomerular filtration rate, GFR)", "treatment with angiotensin II receptor blockers (ARBs) or angiotensin-enzyme-converting inhibitors (ACE-I)".

2) Ancestors of the outcome were selected based on available evidence [2-9]: "Atrial fibrillation (AF)/atrial flutter (AFL) onset", "hypertension", "left and right atrial size", "ejection fraction", "sex", "diabetes mellitus", "coronary artery disease", "AF rate" (index of atrial remodeling), "*de novo* AF", "heart rate", "age", "mitral regurgitation" and "NYHA".

3) Baseline variables that differed significantly between patients who received IV potassium/magnesium and those who did not. These variables were "NT-proBNP level", "heart failure", "troponin T level", "C-reactive protein level", "administration of diazepam", "rate control medication" and "amount of crystalloid fluid".

Based on the relational assumptions made (see eFigure 1), the following role allocation resulted for the following covariables:

1) ***Confounders*** *(ancestors of the exposure and the outcome): "Baseline potassium level", "baseline magnesium level", "heart failure", "onset of AF", "C-reactive protein level", "heart rate", "rate control medication",*

© 2022 Cacioppo C et al. *JAMA Network Open.*

“kidney disease/GFR”, “pH”, “diabetes mellitus”, “hypertension”, “coronary artery disease”, “treatment with ARBs or ACE-I”.

2) ***Mediators*** *(descendant of the exposure and ancestor of the outcome): “Effective potassium level”, “Effective magnesium level”.*

3) ***Colliders*** (a descendant of both the exposure and the outcome): none.

© 2022 Cacioppo C et al. *JAMA Network Open.*

eFigure 1. Directed acyclic graph

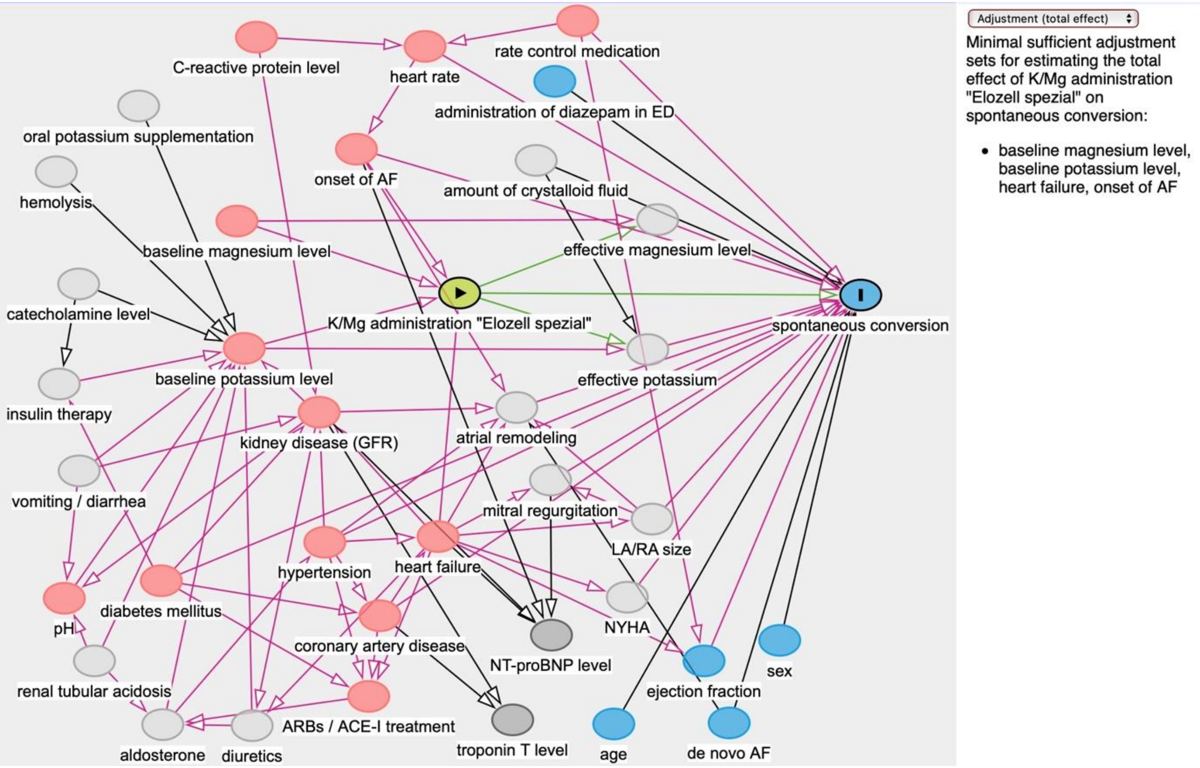

- *The green variable is the exposure.*
- *Red variables are potential confounders (ancestors of the exposure and the outcome). Red arrows mark "biasing paths" that need adjustment. A minimal sufficient adjustment set is thus defined as a set where no uncontrolled biasing paths remain.*
- *Blue variables are ancestors of the outcome.*
- *Dark gray variables are neither confounders, mediators nor colliders.*
- *Light gray variables are "unobserved" (unknown).*
- *The effect of baseline potassium level on the outcome was analyzed using stratification.*

For the total effect of potassium/magnesium administration on outcome, the following minimal sufficient variable adjustment set was determined: "baseline potassium level", "baseline magnesium level", "heart failure", and "onset of AF" (See eFigure 1 top right and eFigure 2). The baseline effect estimate of potassium/magnesium administration on spontaneous conversion to sinus rhythm conditional on this variable adjustment set was 1.39 (risk ratio, 95% CI 1.09–1.76).

© 2022 Cacioppo C et al. *JAMA Network Open.*

eFigure 2. Minimal sufficient variable adjustment set after controlling for biasing paths.

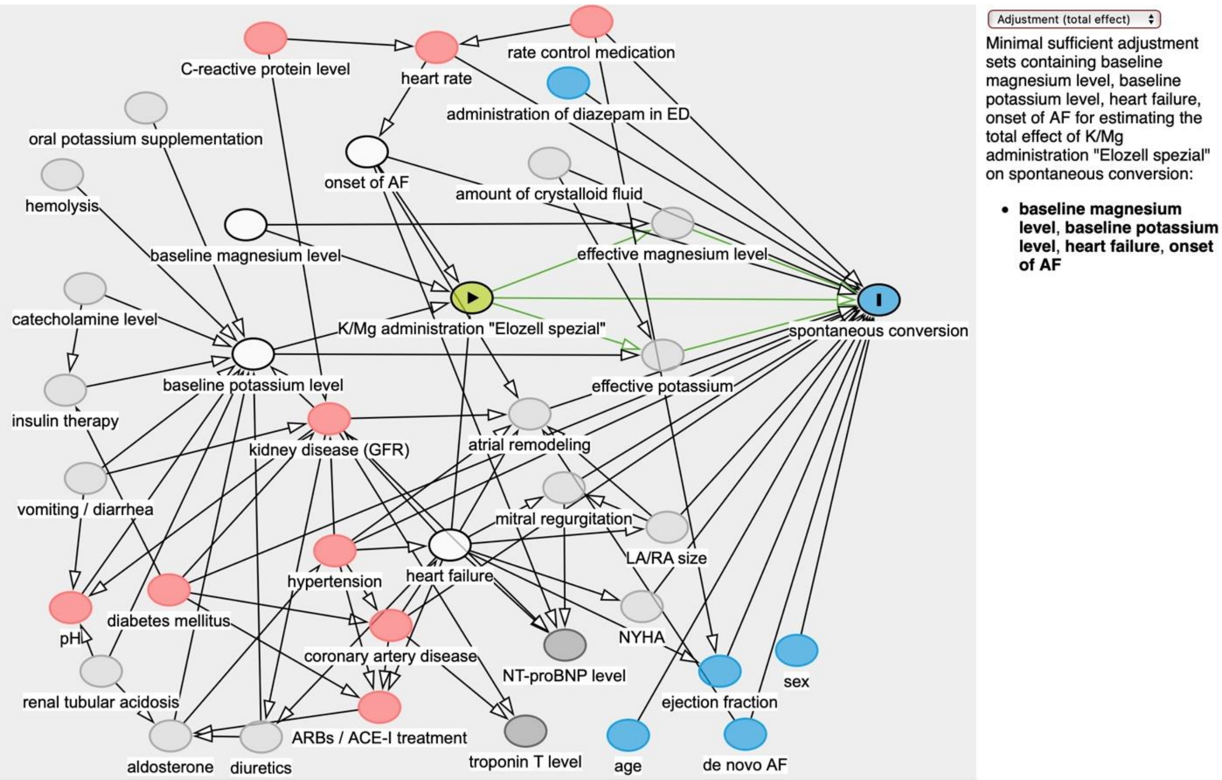

Change-in-estimate procedures:

Based on the relationship assumptions and role allocations laid out in the graph (eFigure 2), we performed a change-in-estimate procedure using a collapsible estimator (risk ratio) to make further variable adjustments. We estimated the change in the baseline estimate for the effect of potassium/magnesium administration when adjusted for other possibly related (confounding) variables (eTable 1).

The implied add-one pattern for the graph had no meaningful change (defined as ± 10% change of the risk ratio) when adding one of the following variables: “C-reactive protein”, “heart rate”, “rate control medication”, “administration of diazepam in the ED”, “glomerular filtration rate”, “hypertension”, “pH”, “diabetes mellitus”, “coronary artery disease”, “treatment with ARBs or ACE-I”, “sex”, “age”, “*de novo* AF” and “baseline calcium level”. The implied minus-one pattern for the minimal variable adjustment set was a change when removing “baseline potassium level”, “baseline magnesium level”, “heart failure”, or “onset of AF”.

As shown in eTable 1, adding or subtracting these variables did significantly change the effect size only for “baseline potassium level”.

© 2022 Cacioppo C et al. *JAMA Network Open.*

eTable. Variables included in the change-in-estimate procedure

**MINUS ONE**		**ADD ONE**	
**Variables**	**Risk Ratio of the main effect**	**Variables**	**Risk Ratio of the main effect**
Baseline K+ level	1.63	CRP	1.41
Baseline Mg2+ level	1.44	Heart rate	1.38
Heart failure	1.39	Rate control medication	1.37
Onset of AF	1.52	Diazepam in ED	1.42
		GFR	1.40
		Hypertension	1.38
		pH	1.36
		Diabetes mellitus	1.39
		Coronary artery disease	1.39
		ARB/ACE-I treatment	1.38
		Sex	1.38
		Age	1.44
		de novo AF	1.33
		Baseline Ca2+ level	1.32

© 2022 Cacioppo C et al. *JAMA Network Open.*

eFigure 3. Changes in baseline estimate by subtracting or adding possibly confounding variables

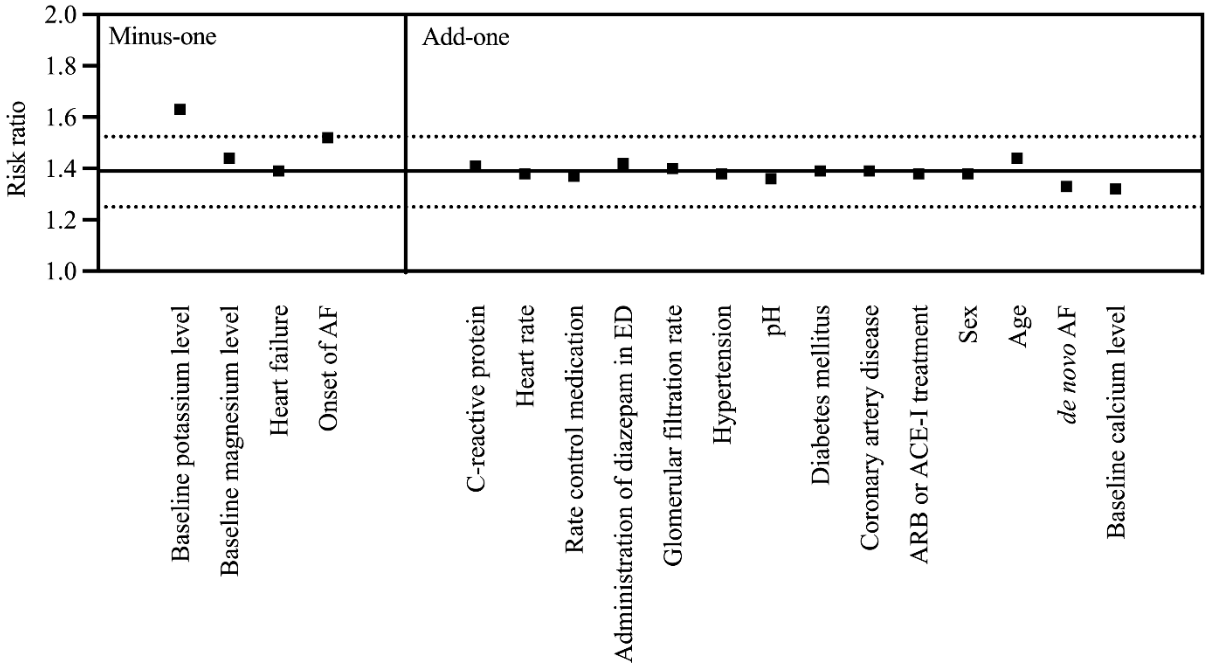

In a change-in-estimate procedure, each variable in the original set of variables was excluded separately (minus one). Then, several variables that were not in the variable set were added separately: C-reactive protein, heart rate, intake of rate control medication, administration of diazepam in ED, glomerular filtration rate, hypertension, pH, diabetes mellitus, coronary artery disease, ARB or ACE-I treatment, sex, age, de novo *AF, baseline calcium level. The solid horizontal line represents the effect estimate of the original model, the dashed horizontal lines indicate the thresholds for meaningful changes in the estimate. The squares indicate the change in the estimate by subtracting (left) or adding (right) individual variables.*

According to the procedure, the most parsimonious multivariable regression model only needs to adjust for baseline potassium levels. However, because adjustment for the onset of atrial fibrillation, comorbidities, and medications is relevant from a clinical perspective, we chose a set of variables that we considered interesting from a clinical point of view and present the multivariable adjusted effect in the manuscript. Furthermore, because the effect of exposure (potassium/magnesium administration) on outcome was not constant across levels of potassium and magnesium and duration of onset, we handled these variables by stratification and modeled them as categorized variables (K+ <3.5, 3.5-3.99, 4.0-4.49, ≥4.5 mEq/L; Mg2: <1.87, 1.87-2.00, 2.01-2.12, ≥ 2.13 mg/dL, onset < 48h, ≥48h and unknown), as presented in the manuscript.

© 2022 Cacioppo C et al. *JAMA Network Open.*

eReferences:

1. Textor J, van der Zander B, Gilthorpe MS, Liskiewicz M, Ellison GT. Robust causal inference using directed acyclic graphs: the R package 'dagitty'. *Int J Epidemiol*. Dec 1 2016;45(6):1887-1894. doi:10.1093/ije/dyw341
2. Danias PG, Caulfield TA, Weigner MJ, Silverman DI, Manning WJ. Likelihood of spontaneous conversion of atrial fibrillation to sinus rhythm. *J Am Coll Cardiol*. Mar 1 1998;31(3):588-92. doi:10.1016/s0735-1097(97)00534-2
3. Abadie BQ, Hansen B, Walker J, et al. Likelihood of Spontaneous Cardioversion of Atrial Fibrillation Using a Conservative Management Strategy Among Patients Presenting to the Emergency Department. *Am J Cardiol*. Nov 15 2019;124(10):1534-1539. doi:10.1016/j.amjcard.2019.08.017
4. Choudhary MB, Holmqvist F, Carlson J, Nilsson HJ, Roijer A, Platonov PG. Low atrial fibrillatory rate is associated with spontaneous conversion of recent-onset atrial fibrillation. *Europace*. Oct 2013;15(10):1445-52. doi:10.1093/europace/eut057
5. Cotter G, Blatt A, Kaluski E, et al. Conversion of recent onset paroxysmal atrial fibrillation to normal sinus rhythm: the effect of no treatment and high-dose amiodarone. A randomized, placebo-controlled study. *Eur Heart J*. Dec 1999;20(24):1833-42. doi:10.1053/euhj.1999.1747
6. Dell'Orfano JT, Patel H, Wolbrette DL, Luck JC, Naccarelli GV. Acute treatment of atrial fibrillation: spontaneous conversion rates and cost of care. *Am J Cardiol*. Mar 1 1999;83(5):788-90, A10. doi:10.1016/s0002-9149(98)00993-x
7. Geleris P, Stavrati A, Afthonidis D, Kirpizidis H, Boudoulas H. Spontaneous conversion to sinus rhythm of recent (within 24 hours) atrial fibrillation. *J Cardiol*. Feb 2001;37(2):103-7.
8. Lindberg S, Hansen S, Nielsen T. Spontaneous conversion of first onset atrial fibrillation. *Intern Med J*. Nov 2012;42(11):1195-9. doi:10.1111/j.1445-5994.2011.02600.x
9. Tejan-Sie SA, Murray RD, Black IW, et al. Spontaneous conversion of patients with atrial fibrillation scheduled for electrical cardioversion: an ACUTE trial ancillary study. *J Am Coll Cardiol*. Nov 5 2003;42(9):1638-43. doi:10.1016/j.jacc.2003.06.008

© 2022 Cacioppo C et al. *JAMA Network Open.*
